# Eosinophilic Granulomatosis with Polyangiitis: An Overview

**DOI:** 10.3389/fimmu.2014.00549

**Published:** 2014-11-03

**Authors:** Andrea Gioffredi, Federica Maritati, Elena Oliva, Carlo Buzio

**Affiliations:** ^1^Unit of Nephrology, University Hospital of Parma, Parma, Italy

**Keywords:** eosinophilic granulomatosis with polyangiitis, vasculitis, eosinophils, vascular diseases, ANCA-associated vasculitis

## Abstract

Eosinophilic granulomatosis with polyangiitis (EGPA) is a multisystemic disorder, belonging to the small vessel anti-neutrophil cytoplasmic antibody (ANCA)-associated vasculitis, defined as an eosinophil-rich and necrotizing granulomatous inflammation often involving the respiratory tract, and necrotizing vasculitis predominantly affecting small to medium-sized vessels, associated with asthma and eosinophilia. EGPA pathogenesis is not well known: HLA-DRB1*04 and *07, HLA-DRB4 and IL10.2 haplotype of the IL-10 promoter gene are the most studied genetic determinants. Among the acquired pathogenetic factors, the exposure to different allergens, infections, vaccinations, drugs, and silica exposure have been involved. Eosinophils are the most characteristic cells in EGPA and different studies have demonstrated their role as effector and immunoregulatory cells. EGPA is considered as a disease with a prevalent activation of the Th-2 cellular-mediated inflammatory response and also humoral immunity plays an important role. A link between B and T inflammatory responses may explain different disease features. EGPA typically develops into three sequential phases: the allergic phase, distinguished by the occurrence of asthma, allergic rhinitis, and sinusitis, the eosinophilic phase, in which the main pathological finding is the eosinophilic organ infiltrations (e.g., lungs, heart, and gastrointestinal system), and the vasculitic phase, characterized by purpura, peripheral neuropathy, and constitutional symptoms. ANCA (especially pANCA anti-myeloperoxidase) are present in 40–60% of the patients. An elevation of IgG4 is frequently found. Corticosteroids and cyclophosphamide are classically used for remission induction, while azathioprine and methotrexate are the therapeutic options for remission maintenance. B-cell depletion with rituximab has shown promising results for remission induction.

## Introduction and Epidemiology

Eosinophilic granulomatosis with polyangiitis (EGPA) is a multisystemic disorder, belonging to the small vessel anti-neutrophil cytoplasmic antibody (ANCA)-associated vasculitides (AAVs). According to the 1994 Chapel Hill consensus conference (CHCC), EGPA is defined as an eosinophil-rich and granulomatous inflammation often involving the respiratory tract, and necrotizing vasculitis predominantly affecting small to medium-sized vessels, associated with asthma and eosinophilia. Formerly known as “Churg–Strauss syndrome,” this eponym has been replaced during the 2012 Revised International CHCC, with the aim of focusing on the histopathology of the disease (1). Unlike in the 1990 American College of Rheumatology classification criteria and the former CHCC, the CHCC 2012 has reported for the first time that ANCA are found in EGPA, especially in patients with glomerulonephritis. This reflects some of the newest evidences of the distinction of two EGPA subsets, depending on the presence or the absence of ANCA (Table 1) (2).

**Table 1:** **Diagnostic criteria, classification, and nomenclature of eosinophilic granulomatosis with polyangiitis during the last 20 years**.

Lanham diagnostic criteria (1984)^a^	American College of Rheumatology classification criteria (1990)^b^	Revised International Chapel Hill consensus conference nomenclature of vasculitides (2012)
Asthma	Asthma	Eosinophil-rich and necrotizing granulomatous inflammation often involving the respiratory tract, and necrotizing vasculitis predominantly affecting small to medium vessel, and associated with asthma and eosinophilia. ANCA is more frequent when glomerulonephritis is present.
	Eosinophilia (>10% of total WBC)	
Blood eosinophilia >1500/mm^3^ or >10% of total WBC	Neuropathy	
	Pulmonary infiltrates non-fixed	
Evidence of vasculitis involving two or more organs	Paranasal sinus abnormalities	
	Extravascular eosinophils	

*^a^All three criteria must be met for a diagnosis of EGPA*.

*^b^The presence of four or more of these six criteria yielded a sensitivity of 85% and a specificity of 99.7% for the classification of vasculitis as EGPA*.

*WBC, white blood cells*.

Eosinophilic granulomatosis with polyangiitis incidence in Europe is 0.5–6.8 new cases/year per million populations, whereas its prevalence is 10.7–13 cases per million populations. It mostly affects subjects between 40 and 60 years old and the mean age at diagnosis is 48 years (3).

## Pathogenesis

**Figure 1:**
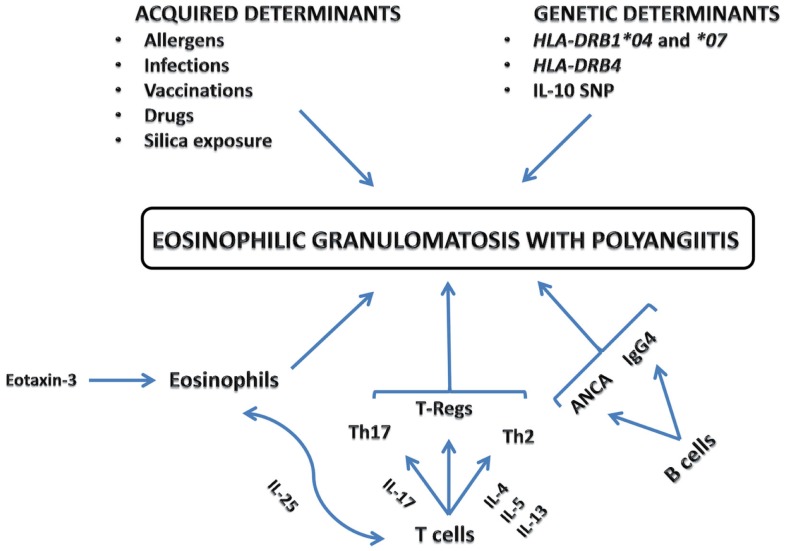
**Eosinophilic granulomatosis with polyangiitis pathogenesis**.

Eosinophilic granulomatosis with polyangiitis pathogenesis is not well known. The disease is probably the result of a complex interaction in which genetically and environmental factors lead to an inflammatory response whose principal players are eosinophils, T, and B lymphocytes (2) (Figure 1).

### Genetic determinants

Eosinophilic granulomatosis with polyangiitis is an HLA-associated disease (4). It has been proven that it is associated with HLA-DRB1*04 and *07 (5) and with *HLA-DRB4* (6). This contraction of the class II HLA repertoire suggests a strong CD4^+^ T lymphocyte activation, possibly triggered by allergens or antigens.

It has been also investigated the presence of single nucleotide polymorphisms (SNP) of the gene, which encodes interleukin (IL)-10, an important molecule for the activation of the Th-2 pathway; EGPA ANCA-negative subset has been associated with the IL10.2 haplotype of the IL-10 promoter gene, a condition, which leads to an increased production of IL-10 (7). This is apparently in line with EGPA pathogenesis, which is characterized by an increased Th-2 response and an increase in IgG4 levels, both of which seem to be mediated by IL-10.

### Acquired determinants

Some environmental triggers have been identified: the exposure to different allergens, infections, vaccinations could trigger the disease. Drugs may also have a pathogenetic role and, among these, the leukotriene receptor antagonists are the most frequently involved more often used as steroid-sparing agents for asthma, their key role in triggering EGPA is still uncertain (8). More recently, also the recombinant anti-IgE monoclonal antibody omalizumab used in patient with asthma has been considered as an EGPA trigger (9–11). According to the most reliable hypotheses, both LTRA and anti-IgE antibody may be involved in EGPA pathogenesis simply unmasking the disease, due to the delayed use of steroids.

A recent review has shown the possible pathogenetic influence of silica exposure in AAVs, including EGPA (12).

### Eosinophils

The role of the eosinophils is still uncertain in EGPA but different studies have demonstrated the cytotoxic (13, 14) and pro-coagulant (15, 16) properties of this cell type, which may result in the development of cardiovascular and cerebrovascular complications in patients with any type of hypereosinophilic syndromes including EGPA. Although they are usually considered to be effector cells, they may act as immunoregulatory cells (2): indeed, a cross-talk between T-lymphocytes and eosinophils has been pointed out. In a recent study, high concentrations of IL-25 have been detected in the sera of EGPA patients; eosinophils are the main source of IL-25, which induces T-cells to produce cytokines that stimulate Th-2 and, at the same time, eosinophilic responses (17).

### T-lymphocytes

It has been demonstrated that T-lymphocytes have an important role in the EGPA pathogenesis. T-cells are present in the most of the organ lesions and in some of them, like peripheral neuropathy, they represent the main component. Moreover, serum levels of T-cell activation markers, like IL-2r, are increased during the active phase of the disease (18). T-cells receptors show a restricted repertoire suggesting oligoclonal expansion (19), which is in line with the hypothesis of an antigen-driven disease. Clonal restricted effector CD8^+^ lymphocytes with a proinflammatory profile have been recently described in patients with EGPA (20).

Specifically, EGPA is considered as a disease with a prevalent activation of the Th-2 pathway. In keeping with this view, it has been demonstrated that tissue infiltrates in patients with EGPA are rich in T-cells with Th-2 makers such as CD294. Furthermore, EGPA patients CD4^+^ T-cells are able to produce, *in vitro*, high concentrations of IL-4, IL-5, and IL-13, molecules that hallmark the Th-2 immunoresponse.

High-blood concentrations of IL-17 have been found in patients with EGPA, a finding, which suggests that the involvement of Th17 lymphocytes into EGPA pathogenesis; indeed, these lymphocytes are involved in the pathogenesis of other autoimmune diseases (2).

Finally, reduced levels of regulatory CD4^+^ T-cells (Tregs) have been discovered in EGPA patients (21, 22). Tregs usually have a protective role toward the development of autoimmune diseases. Lower numbers of Tregs were found in active EGPA patients than in patients with asthma or with chronic eosinophilic pneumonia; additionally, the percentages of circulating Tregs were lower in active than quiescent EGPA (2).

### B-lymphocytes

The role of the humoral immunity in EGPA seems to be less relevant as compared to other autoimmune diseases. Despite this, EGPA patients often show an abnormal humoral response. ANCA are found in about 40% of patients with EGPA, they are characterized by a perinuclear pattern (pANCA) at the immunofluorescence assay and are directed against the neutrophil myeloperoxidase (MPO), as revealed by ELISA. Their pathogenetic role and their potential harmful effect is still matter of debate. Even though animal models and *in vitro* studies have shown a pathogenic role of the anti-MPO antibodies (23), their role in causing organ damage in EGPA is still unknown.

A substantial number of patients show an increased IgG4 blood levels. In a recent analysis of 46 EGPA patients, IgG4 levels correlated with the number of disease manifestations and the Birmingham vasculitis activity score (BVAS). Furthermore, serum IgG4 levels paralleled the disease course as they normalized during remission. The skewed IgG4 response is likely due to the enhancing effects of the Th-2 cytokines IL-4, IL-5, and IL-13 (24).

### Cytokines and chemokines

Regarding chemotaxis, eotaxin-3 (CCL26), a chemokine, which attracts eosinophils in the sites of inflammation, apparently has a key role into EGPA pathogenesis. Two different studies reported that eotaxin-3 was highly elevated in serum samples of active EGPA patients and correlated highly significantly with eosinophil counts, total immunoglobulin E (IgE) levels, and acute-phase parameters. Immunohistochemical analysis revealed strong expression of eotaxin-3 in endothelial and inflammatory cells in affected tissues of active EGPA patients (25, 26).

Also, CCL17, another Th-2 chemokine, seems to be present into both tissues infiltrates and patients’ sera (27).

Some recent studies have demonstrated that EGPA patients’ T-cells produce, after stimulation *in vitro*, a large amount of interferon-γ (INF-γ), a cytokine, which boosts Th-1 immune response (28).

The hypothesis of a cross-talk between humoral and cell-mediated immunity and eosinophils is still the object of different pathogenetic studies.

## Clinical Features

Eosinophilic granulomatosis with polyangiitis mainly affects patients with asthma (often developed in the adult age), sinusitis, allergic rhinitis, and nasal polyposis (Table 2) (29).

**Table 2:** **Main clinical features in eosinophilic granulomatosis with polyangiitis and their prevalences**.

Clinical features	Prevalence (%)	Reference
Mean age at diagnosis (years)	50 ± 16	Comarmond et al. (30)
Asthma	91–100	Comarmond et al. (30); Sablé-Fourtassou et al. (31)
Ear, nose, and throat involvement	48–75	Comarmond et al. (30); Bacciu et al. (32)
Neuropathy	55–72	Comarmond et al. (30); Sablé-Fourtassou et al. (31)
Pulmonary involvement	65–91	Sablé-Fourtassou et al. (31); Comarmond et al. (30)
Cutaneous involvement	40–52	Comarmond et al. (30); Sablé-Fourtassou et al. (31)
Renal involvement	27	Sinico et al. (33)
Cardiac involvement	27–35	Comarmond et al. (30); Sablé-Fourtassou et al. (31)
Gastrointestinal involvement	23–32	Comarmond et al. (30); Sablé-Fourtassou et al. (31)
Central nervous system involvement	5–9	Comarmond et al. (30); Sablé-Fourtassou et al. (31)
ANCA positivity	38	Sinico et al. (34)
pANCA positivity	74 of all ANCA^+^ patients	Sinico et al. (34)

*ANCA, anti-neutrophil cytoplasmic antibody*.

Eosinophilic granulomatosis with polyangiitis typically develops into three sequential phases, marked by a progression of the main symptoms. The first phase, also called prodromic or allergic, is most common in the second or third decade and it is distinguished by the occurrence of asthma, allergic rhinitis, and sinusitis. Subsequently, the eosinophilic phase develops the main pathological findings of this phase are the raise in the peripheral eosinophilic count and the eosinophilic organ infiltrations, especially in lungs, heart, and gastrointestinal system. The third phase is the vasculitic one during this last phase, the patient suffers from the consequences of a necrotizing vasculitis (e.g., purpura, peripheral neuropathy), generally associated with vascular or extravascular granulomatosis and constitutional symptoms like fever, malaise, and weight loss (35).

Eosinophilic granulomatosis with polyangiitis is a multisystemic disease. One of the most frequently involved sites is the respiratory system asthma has a prevalence of about 95% (36). Pulmonary eosinophilic infiltrates may be present and their biopsy is often highly informative for the histopathologic diagnosis (37).

The otorhinolaryngoiatric system is also frequently involved nasal polyposis is one of the conditions, which lead patients to undergo repeat surgery. Allergic rhinosinusitis, epistaxis, and neurosensory hearing loss are other common features (32).

Cardiac involvement may be represents the most harmful manifestation of EGPA characterized by myocardial infarction, pericarditis, or congestive heart failure, it is the main cause of death (30, 36, 38).

Among the most frequent skin manifestations, subcutaneous nodules, and purpura (especially involving the legs) represent a clinical hallmark of the vasculitic phase, a skin biopsy of purpuric lesions generally shows a leukocytoclasic vasculitis (39, 40).

Although less frequent than the other two AAVs, renal involvement occurs in about 25% of the patients and the most typical expression is pauci-immune crescentic glomerulonephritis with a high range of clinical features, from isolated urinary abnormalities (proteinuria, hematuria) to rapidly progressive glomerulonephritis. Kidney involvement is a bad prognosis factor for patients with EGPA (33).

Peripheral neuropathy, either sensory or motor or sensory-motor, affects a large portion of the patients; mononeuritis multiplex, with axonal damage, usually unilateral and asymmetric, is the most characteristic manifestation of peripheral nervous system involvement. Patients report paresthesia and pain in the affected areas (peroneal, tibial, ulnar nerve), especially during the vasculitic stage of the disease (41).

In the gastrointestinal system, the vasculitic phase may be preceded by an eosinophilic gastroenteritis with abdominal pain, diarrhea, and intestinal bleeding (42).

In the 30–40% of the patient, there can be diffuse lymphadenopathy, frequently affecting axillary and cervical lymph nodes (43).

The most frequent laboratory findings in EGPA patients is marked hypereosinophilia, frequently between 5000 and 9000 eosinophils/μL [at least >1500 eosinophils/μL or >10% of the total white blood cells, according to Lanham criteria (44)], this is one of the most common signs of EGPA (36). An increase in non-specific inflammatory markers (ESR, CRP) is often found (36). The role of the complement is still uncertain. ANCA are present approximately in 40–60% of the patients; pANCA (perinuclear) is the prevalent pattern, with antibody specificity for MPO (33, 34, 45).

All these clinical manifestations and laboratory features could be frequently gathered into two patterns: the *vasculitic* and ANCA-positive phenotype, characterized by manifestations resulting from small and medium-sized vessel vasculitis (e.g., purpura, mononeuritis multiplex, glomerulonephritis) and the *eosinophilic*, ANCA-negative phenotype, in which the organ is damaged mainly by an eosinophilic infiltration (e.g., pulmonary infiltrates, cardiomyopathy) (2). These findings may have pathogenetic implications, as they suggest that ANCA, as observed in MPO-ANCA mouse models, mediate vasculitis in EGPA as well; however, there are no animal models of EGPA. In addition, the ANCA-positive and ANCA-negative subsets are not clearly separated, as overlapping manifestations occur very frequently.

## Histopathology

The main histological findings in EGPA are the extravascular granulomas, small and medium-sized vessels vasculitis, and the eosinophilic infiltrates.

Interstitial and vascular granulomas are composed by eosinophilic necrotic matrix surrounded by giant cells and palisading lymphocytes. The vasculitic process affects mainly small and medium vessels (especially small arteries) and is characterized by fibrinoid necrosis of the vessel wall associated or not with granuloma or eosinophilic infiltrates (46, 47). It is difficult to find all these features together, which makes the histological diagnosis sometimes challenging (48). In addition, specific disease manifestations often show specific histopathological features; for example, purpura is caused by a leucocytoclastic vasculitis (eosinophilic infiltration or fibrinoid necrosis is frequently absent) and alveolar hemorrhage depends on an alveolar capillaritis (without granuloma) (36). Furthermore, glomerulonephritis (33) and peripheral neuropathy frequently lack eosinophilic infiltrates. Gastrointestinal biopsies reveal eosinophilic tissue infiltration and histological signs of mesenteric vessel vasculitis, which may induce bowel ischemia (42).

Cardiac involvement may show coronary vasculitis, myocardial granuloma, eosinophilic endomyocarditis, and pericarditis (36).

## Differential Diagnosis

Different conditions have to be considered in the differential diagnosis, mainly eosinophilic and vasculitic diseases.

Parasitic infections as well as hypersensitivity reactions (e.g., to drugs) must be excluded. The hypereosinophilic syndrome (HES) is characterized by persistent eosinophilia and organ involvement without a reason, which can explain hypeosinophilia. Cardiac and pulmonary manifestations are analog to those of EGPA patients but subjects with HES usually do not have asthma or vasculitic complication like purpura or glomerulonephritis; furthermore, ANCA are absent in HES (49). A recent revised classification of HESs has focused on the pathogenesis of many hypereosinophilic disorders: myeloproliferative and lymphocytic forms of HES should be excluded in all patients. Particularly, Fip1-like-1(FIP1L1)/platelet-derived growth factor receptor α (PDGFRA) fusion genes must be investigated (50).

Broncho-pulmonary allergic aspergillosis may mimic pulmonary involvement in EGPA: differential diagnosis is helped by finding *Aspergillus* spp at bronchoscopy lavage or dosing *Aspergillus fumigatus* specific serum IgE, which are pathognomonic of allergic aspergillosis (51).

Acute eosinophilic pneumonia is featured by pulmonary infiltrates and bronchoscopy lavage rich in eosinophils but usually originates as an acute illness with fever and dyspnea, without peripheral eosinophilia or other organ involvement.

Chronic eosinophilic pneumonia diagnosis is more insidious. Patients may present with asthma, peripheral eosinophilia, and constitutional symptoms. The absence of other organ manifestations and the negativity of ANCA may help to differentiate chronic eosinophilic pneumonia from EGPA (52).

Eosinophilic granulomatosis with polyangiitis must be distinguished from the other AAVs. Granulomatosis with polyangiitis (GPA) may mimic particular aspects of EGPA, especially in those patients, which present peripheral eosinophilia, the ANCA specificity (cANCA PR3-specific, in GPA) and the presence, in GPA, of pulmonary cavitated nodules associated with nasal crusting and nasal and paranasal sinuses erosion, allow clinicians to differentiate the two vasculitides.

Although microscopic polyangiitis (MPA) could be also characterized by pANCA with MPO specificity, it rarely shows peripheral eosinophilia, nodules, or eosinophilic pulmonary infiltrates (48).

Finally, EGPA must be differentiated from IgG4-related disease (IgG4-RD), which may present with allergic manifestations, blood eosinophilia, pulmonary infiltrates, and sinusitis. However, tissue biopsies in patients with IgG4-RD show fibrosis and obliterative phlebitis, without vasculitis or eosinophilic granulomas (53).

In our center experience, first level examinations include blood tests and, in particular, complete blood cell count, ESR, CRP, immunoglobulins with their subclasses (especially IgG subclasses), rheumatoid factor, ANCA, eosinophil cationic protein (ECP), serum B12 levels (elevated in myeloproliferative neoplasms), and a screening of renal function and urinalysis. Detection of FIP1L1/PDGFRA fusion genes and stool cultures for ova and parasite examination must be done in the early stages of diagnosis. ANCA are thought to be useful in the differential diagnosis between EGPA and other (especially infectious and hematological) eosinophilic disorders. Likewise, finding fusion genes clearly points toward a diagnosis of myeloproliferative HES. The differential diagnosis with lymphocytic forms of HES is more challenging, as most laboratories do not perform clonal analysis of circulating lymphocyte subsets or their intracellular cytokine production, which could be helpful in these conditions.

Second level examinations include imaging studies such as lung and facial computed tomography (CT), as well as functional studies such as electromyography.

Finally, kidney biopsy and a bronchoscopy with bronchoalveolar lavage are reserved for those patients with severe (and often rapidly progressive) clinical manifestations.

## Treatment and Outcome

Eosinophilic granulomatosis with polyangiitis treatment is a matter of debate because of the lack of large-scale, randomized controlled trials. The five factors score (FFS) may be a guide for clinicians, this score assigns one point to each of the following items, namely, gastrointestinal involvement, CNS involvement, cardiac involvement, proteinuria >1 g/24 h and serum creatinine >141 μmol/L (35). Patients with poor prognosis factors (FFS ≥1) are often treated with both glucocorticoids (classically prednisone at dosage of 1 mg/kg of total body weight/day with a maximum dosage of 75 mg/day, for 1 month and then tapered) and cyclophosphamide (CYC, 2 mg/kg of total body weight/day), while the typical approach for patients with a better prognosis (e.g., FFS of 0) is glucocorticoid therapy alone (54). Recently, a revised FFS has been proposed an age over 65 years, cardiac symptoms, gastrointestinal involvement, renal insufficiency (serum creatinine >150 μmol/L) and absence of ear, nose, and throat manifestations have been pointed out as predictors of 5-year mortality (55).

Classically, used therapies in EGPA remission maintenance are azathioprine or methotrexate (56).

Although primarily used for GPA, the BVAS, a clinical index of disease activity (57), might be useful to better decide when to stop therapy with CYC and introduce maintenance therapy like azathioprine or methotrexate.

Cyclophosphamide toxicity has long been known (58) and, based on our center experience, we recommend not to exceed the dose of 10–15 g of CYC (including both oral and pulse medications). On the other hand, too-short duration of CYC administration has been associated with more relapses (59).

Azathioprine too requires a constant monitoring of liver function, due to the drug-related hepatotoxicity (60).

B-cell depletion adjunct therapy with rituximab has shown promising results for remission induction (61–67).

Interleukin-5, a major survival factor for eosinophils, has been targeted in patients with EGPA using the monoclonal antibody mepolizumab. Use of mepolizumab in refractory cases (68, 69) and steroid-dependent patients (70) has given positive results but EGPA manifestations recurred on drug cessation.

On the assumption of its inhibitory effects on the eosinophil degranulation, interferon-alpha therapy has been tried with positive results in refractory patients, but the severe drug-related toxicity has greatly limited its use (71, 72).

Plasmapheresis may be an adjunctive therapy particularly in patients with rapidly progressive glomerulonephritis, peripheral neuropathy, or alveolar hemorrhage (2).

Eosinophilic granulomatosis with polyangiitis outcomes are well represented in a retrospective study of 383 EGPA patients in the French Vasculitis Study Group cohort. Vasculitis relapse occurred in 97 patients (25.3%), while 72 additional patients experienced asthma flares, sinusitis, and/or increased eosinophilia. Of the 383 patients, 45 (11.7%) died and the major cause of death was attributed to cardiac events. Five-year and 10-year survival rates were, respectively, 88.9 and 78.6%. Vasculitis relapse-free survival rate at 5 years was 64.8%, while at 10 years was 54.4%. ANCA positivity and cutaneous signs were independent predictors of relapse (30).

Another recent analysis of EGPA patients’ long-term follow up has demonstrated that the outcome of EGPA is good with respect to mortality. According to the analysis of 118 patients with EGPA (enrolled in two prospective trials), 108 (91.5%) patients achieved remission (34 of the 108 achieved long-term remission without relapse) and 12 (10.2%) died (only 5 of them died for EGPA-related causes). During relapses, pulmonary symptoms predominated (81%), followed by ear nose and throat signs (38%) and mononeuritis multiplex (36%) (73).

Finally, in a German cohort of 150 EGPA patients, the analysis of the follow-up of 104 of them has evidenced that 70 patients (67.3%) attained remission after conventional therapies, 21 (14%) suffered from major relapses and 42 (28%) from minor relapses. Twelve patients died 94 ± 16 (mean ± SD) months after diagnosis (74).

## Perspective Future

Despite the great levels of knowledge reached, more has to be done to clarify EGPA pathogenesis, a genome-wide association study (GWAS) will probably help to better understand the genetic determinants of the disease. Besides, the environmental factors like silica or any other occupational exposure (e.g., asbestos) must be studied in depth.

In the future, probably, the distinction between ANCA^+^ and ANCA^−^ small vessels vasculitides will lead to re-define the current classification criteria with a more simplistic view of all the AAVs.

Despite this, clinicians should keep in mind all the distinctive clinical features and differential diagnosis approaches that make EGPA one of the more characteristic and complex AAV.

## Conflict of Interest Statement

The authors declare that the research was conducted in the absence of any commercial or financial relationships that could be construed as a potential conflict of interest.
